# A New Benchmark for Consumer Visual Tracking and Apparent Demographic Estimation from RGB and Thermal Images

**DOI:** 10.3390/s23239510

**Published:** 2023-11-29

**Authors:** Iason-Ioannis Panagos, Angelos P. Giotis, Sokratis Sofianopoulos, Christophoros Nikou

**Affiliations:** 1 Department of Computer Science and Engineering (CSE), University of Ioannina, 45110 Ioannina, Greece; i.panagos@uoi.gr (I.-I.P.); cnikou@uoi.gr (C.N.); 2 Institute for Language and Speech Processing (ILSP), Athena Research and Innovation Center, 15125 Athens, Greece; s_sofian@athenarc.gr

**Keywords:** consumer tracking, demographic-data estimation, target detection, motion prediction, tracklet association, multi-attribute classification, consumer metadata

## Abstract

Visual tracking and attribute estimation related to age or gender information of multiple person entities in a scene are mature research topics with the advent of deep learning techniques. However, when it comes to indoor images such as video sequences of retail consumers, data are not always adequate or accurate enough to essentially train effective models for consumer detection and tracking under various adverse factors. This in turn affects the quality of recognizing age or gender for those detected instances. In this work, we introduce two novel datasets: *Consumers* comprises 145 video sequences compliant to personal information regulations as far as facial images are concerned and *BID* is a set of cropped body images from each sequence that can be used for numerous computer vision tasks. We also propose an end-to-end framework which comprises CNNs as object detectors, LSTMs for motion forecasting of the tracklet association component in a sequence, along with a multi-attribute classification model for apparent demographic estimation of the detected outputs, aiming to capture useful metadata of consumer product preferences. Obtained results on tracking and age/gender prediction are promising with respect to reference systems while they indicate the proposed model’s potential for practical consumer metadata extraction.

## 1. Introduction

Visual tracking of multiple targets, also referred to as multiple object tracking (MOT) [1,2], since the target can be any moving object or entity, is a well-investigated computer vision task. Actually, the goal is to detect one or more targets in a time-variate scene and then obtain their trajectories in terms of following their tracklets, for a given video sequence. This is completed by associating newly detected instances with current ones. Typically, the association part assumes a prediction task whose aim is to favor the most possible correspondence among detections of consecutive frames for a given target. When the targets of interest are real people, resulting detections from this procedure are usually post-processed so as to extract useful information related, for instance, with their age or gender. To that end, novel classification and regression algorithms have been proposed, mainly relying on representation learning from facial attributes [3]. The real interest in this framework of distinct computer vision tasks lies at the adaptation flexibility of each proposed system, to the particularities of a specific visual tracking task (e.g., consumer tracking), where the results need to be combined with further processing steps so as to yield meaningful target insights [4].

### 1.1. Multiple Object Tracking (MOT)

Multi-object tracking (MOT) has been widely studied in recent years and a large variety of tracking algorithms have emerged, evolving from methods relying on hand-crafted image representations (e.g., graphs [5,6], SIFT, HOG-like features), to deep-learning-based approaches (i.e., graph neural networks [7]) that aggregate information across frames and objects or even combine tracking and detection techniques [8] to perform *joint detection and tracking* [9] so as to improve the tracking performance with multiple frames.

Concerning the motion prediction part, MOT techniques have advanced from standard Kalman filters [10,11] by incorporating (or even replacing them with) recurrent neural networks (RNN) [12] and long short-term memory (LSTM) networks [13] to boost association performance. LSTMs are also adopted in our proposed system in this fashion.

Tracking multiple objects faces many challenges due to the complex nature of the problem which involves several steps. Even when objects are accurately detected, identity preservation for those objects in subsequent frames, or trajectory management (i.e., terminating and re-instantiating trajectories when the tracked targets disappear from, or re-appear in the current frame, respectively), are a few notable challenges that need to be addressed for accurate tracking. Moreover, target overlaps, background objects as well as changes in target movement direction and orientation are additional variables that interfere with trajectory prediction and should not be neglected by a robust MOT system with low bias in its predictions. No to mention that suboptimal results from each step are transferred to the remaining components, significantly affecting the overall result. For example, inadequate detections or incorrect target associations can lead to substantial variance in tracking quality. Hence, recent research progress aims to alleviate potential drawbacks bound to the preliminary parts of the tracking pipeline.

Applications of multiple object tracking are widespread in computer vision. For instance, apart from video surveillance [14] and pedestrian tracking [15,16], which are typical use cases, MOT systems can assist in traffic flow analysis [17,18] or autonomous driving [19] and have even been employed for the tracking of athletes [20].

**Figure 1:**
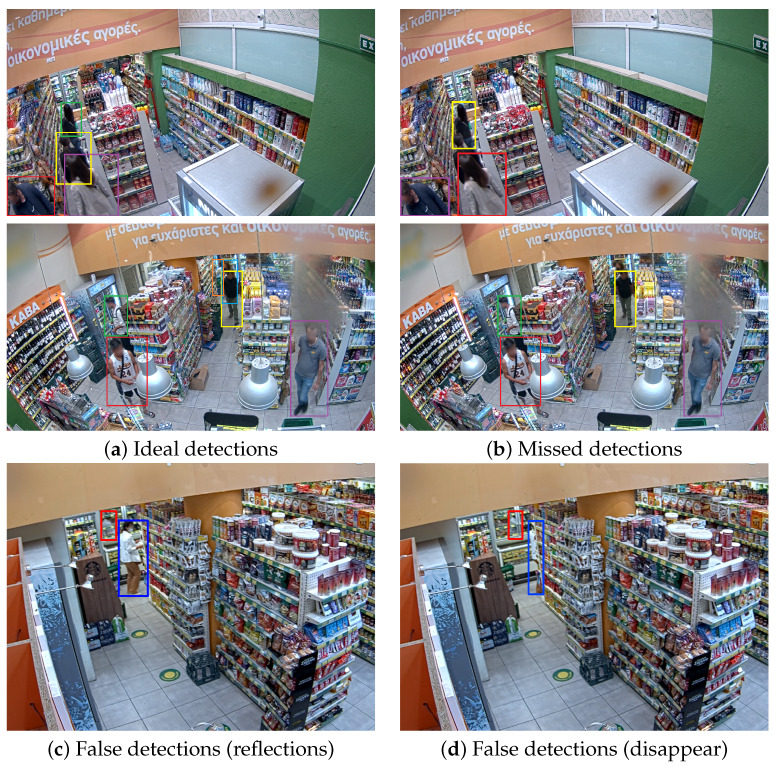
Challenges related to the quality of the multi-object tracking of consumers from indoor camera images in retail shops. (**a**,**b**) Ideal versus standard detection results of occluded consumers. (**c**,**d**) False detections due to reflections (depicted in red color) even after disappearing from the scene. All instances are taken from our *Consumers* dataset.

In this work, we focus on the task of MOT where the objects of interest are retail consumers. In such cases, additional factors that can potentially hamper tracking performance need to be taken into consideration. Concretely, sequences depicting indoor retail locations, such as shop hallways, entail additional challenges due to occlusions from clutter, visual noise in the form of illumination variance from reflections and shadows or uneven lighting conditions, and several others. Some of these challenges are illustrated in Figure 1. As a result, prediction accuracy can deteriorate, especially when instances of multiple consumers might overlap due to their physical positioning or other interactions. In other words, standard tracking methods employed for consumer tracking might miss an occluded target (high variance) or detect something that is not actually there (high bias), and therefore, a specialized approach is necessary.

Apart from those inherent limitations related to visual tracking from video sequences, we should note that existing literature in ‘multi-consumer’ tracking assumes that proposed methods have to cope with images compliant to regulations which determine how personal data are obtained, stored, processed, and shared for research purposes (GDPR regulations). To this end, a fully GDPR-compliant tracking system should make sure that initial data are somehow anonymized in due time, before they are further processed by third-party applications (i.e., metadata extraction from demographic information of tracked targets) or employed for research purposes (i.e., a public image dataset that can be used for various computer vision tasks). For this reason, challenges in consumer visual tracking also entail the data acquisition procedure (secure video capture), as well as their processing and storage in a protected and anonymized format with only supervised or authorized access.

Finally, several techniques follow a multi-modal approach to MOT by exploiting the combinatorial power of multiple input data modalities, such as RGB-thermal, RGB-IR, and other vision-based trackers or even RGB cameras along with sensors. The key idea behind fusing data from multiple modalities lies in increasing tracking performance, since complementary cues might improve the accuracy of detections in highly occluded conditions which could in turn affect the performance during tracklet association [21]. However, modality embedding in a common subspace to extract meaningful representations yet remains an under-explored topic, and typically, a simple early fusion technique is adopted right before feature extraction from the deeper network layers takes place. A similar early fusion approach is also followed in our work when RGB-thermal modalities are employed.

### 1.2. Demographic Estimation (Age and Gender Prediction)

Within this framework of consumer visual tracking, a computer vision task might further focus on recognizing attributes of each detected consumer which can be related to demographic information such as age and gender [22]. This information can be useful for meta-data extraction, related but not limited to consumers’ preferences according to some grouping of their age and/or gender cues. Such information could provide further insight of a consumer’s behavior. In other words, the duration a consumer stares at a specific product, or the time they stand in front of a shelf containing several products of the same type, regardless whether a product is selected or not for purchase, might indicate a preference degree for those products. This in turn allows for a focused product marketing customization.

*Age prediction* can be seen as an actual age recognition problem where the real age of a person is expected as the output of a regression model, not necessarily an integer value but also a real one in an acceptable range (e.g., (18–100)) (https://github.com/openvinotoolkit/open_model_zoo/tree/master/models/intel/age-gender-recognition-retail-0013, accessed on 26 October 2023). In this case, of course, it is assumed that training data with real target ages are available beforehand, while age estimates can be obtained only for those ages for which training images actually include such age values (i.e., child ages cannot be accurately predicted if no child faces exist in the training set).

Age prediction can be also reduced to a multi-class classification problem where a person’s age is classified to a specific age group (i.e., within range (18–30)). Herein, age estimation is considered a more complex task than gender estimation since the target age groups can be more than those of the gender counterpart. More importantly, its inherent complexity can be attributed to the high variability introduced by the aging process, typically reflected by facial attributes [23]. Facial characteristics are generally representative of a person’s age, and thus, comprise the main attention of an age prediction model, compared to the rest of the full body image of the detected consumer instance. Therefore, image features are expected to be more discriminative among different consumer instances, for the part of the image which contains the unique characteristics of a consumer’s face. An age estimation system which deals the problem as a multi-class classification task oriented for audience measurement purposes, is presented in [24]. In addition, a recent comprehensive study [25] overviews the various families of approaches for facial age estimation, mainly focusing on machine-learning-based approaches.

Another variation of the first component of demographic cue recognition is *apparent age estimation* [26] which differentiates from real age inference in terms of assuming ground truth data which derive from the annotator’s estimation when the actual age of each consumer is unknown. In this respect, we can distinguish two main families of approaches. The first one considers multiple ground-truth labels for the same consumer which are clustered into age groups, then fed to proposed age prediction models whose goal is to infer an average age for each tested target [27,28].

The second variation deems that each image (or bounding box detection of consumers) is annotated by a single annotator, and thus, it is expected that inconsistency in ground-truth data will be present among different annotators. However, during age inference, the model is expected to be robust to minor variations in age estimates from different annotators for consumers which are present in two or more images of the test dataset assuming a limited number of known ages are provided during training [29].

Existing literature in apparent age estimation [27] suggests that using apparent labels for training can improve real age estimation rather than entirely training with real ages. Our approach is more closely related to the second category of apparent age estimation wherein no actual consumer’s age is known in advance. For this reason, in our work, the evaluation can be carried out only in a qualitative respect during inference under acceptable performance since the age ranges selected for our in-house dataset are wide enough to allow for apparent age error estimates accrued from different annotators.

*Gender prediction* is a binary classification task which typically assumes facial images as input. Such images are usually obtained by a face detection step based on hand-crafted feature representations or trending convolutional neural networks (CNNs) as feature extractors for face region proposal extraction. The next step is the actual classification of the representation using either traditional machine (SVMs, decision trees) or deep learning algorithms. There are several challenges associated with gender estimation from faces, including variations in facial expression, illumination, occlusion, as well as the presence of facial hair and cosmetics, often tackled with large training corpora of facial images with known gender information. Another precaution consists of data augmentation techniques where initial training images are rotated, scaled, or flipped to increase model robustness to intra-class variability.

Less frequent approaches consider a full-body image as input of the gender classification model usually obtained as a result of an object detection or semantic segmentation task. Once the body is detected, a subsequent step is to extract relevant features that can be used to determine the gender. These features are related to the shape, size, and even texture of the body. More recent techniques deem clothing and accessories as an additional discriminative feature in a multi-attribute classification framework [30] or can combine different modalities such as RGB and thermal imagery [31].

Our approach to gender prediction follows this direction of inferring the estimated gender from a full-body image corresponding to the outcome of the detector of the multi-object tracking component. In a similar fashion to age prediction, training images are bounding boxes labeled with the annotator’s apparent gender estimation, where actual gender information is unknown in the first place.

Gender estimation from full body images entails challenges such as variations in pose, clothing, and accessories, as well as the presence of occlusions and clutter in the background. Not to mention that the face can be entirely occluded in the case of images containing only the rear view of a person. Normally, to overcome these challenges, large and diverse datasets are employed.

Again, it should be noted that apparent demographic estimation is not a trivial task. First, it is not always accurate, since age prediction may be influenced by factors such as genetics, lifestyle, and environmental conditions, while gender estimation can be affected by factors such as cultural and personal beliefs, as well as individual self-expression. Second, in both cases of consumers’ apparent age and gender prediction, it is important to consider the ethical implications of using personal data for marketing purposes and to ensure that the information is collected and processed in a responsible and transparent manner. Especially for the gender information counterpart, its usage in marketing must also comply with respective regulations, including those related to data privacy and anti-discrimination.

### 1.3. Motivation and Contribution

The contributions of our work can be summarized in the following. We introduce two novel datasets of consumers in indoor scenes, the first comprising sequences of video frames for multi-object tracking purposes, whereas the second entails a full-body image dataset that can be utilized for generic attribute or apparent age as well as gender estimation. The datasets feature considerable variability among consumer instances in terms of appearance, age, and gender cues while raw images are also anonymized with respect to data privacy regulations. This anonymity step is carried out using two open access software applications such as deface (https://github.com/ORB-HD/deface accessed on 26 October 2023) and deep privacy (https://github.com/hukkelas/deep_privacy2 accessed on 26 October 2023). The first one actually deforms the region corresponding to the consumer’s face, while the second one replaces the whole face with an artificially generated one based on generative adversarial learning. By these means, the proposed datasets are readily available for evaluating numerous computer vision tasks.

Subsequently, our proposed MOT technique relies on a state-of-the-art tracking-by-detection approach, where standard Kalman filters are replaced with an LSTM network, leading to considerably superior performance as a result of increased accuracy of motion estimation by exploiting the network’s capacity to model long-term dependencies. More specifically, LSTM considers more than one past frames as opposed to its Kalman counterpart (which only considers its previous and current frame), when regressing bounding-box coordinates for each detected target between the current and previous frame. Hence, replacing Kalman filters with LSTM is expected to yield improved tracking performance. Moreover, its real-time application capacity is feasible, since the trajectories of all detected targets are modeled by a single LSTM, and thus, it does not significantly affect running times. Following this MOT rationale a step further, we adopt a simple method for reliable estimation of demographic information directly on the detected full-body images without any facial characteristics. Numerical results validate the effectiveness of our approach for each distinct task. Finally, since both methods are lightweight in terms of computational requirements, they can be seamlessly integrated into a single pipeline that can tackle both tasks simultaneously and runs in real-time speeds, allowing for deployment in practical scenarios. To that end, we have built an end-to-end system for multi-consumer detection, tracking, and *apparent* age/gender prediction, which can facilitate handful consumer meta-data extraction for marketing campaign customization.

The remainder of this article is structured as follows: an overview of related work for the tasks of Multi-Object Tracking (MOT), age estimation, and gender classification is provided in Section 2. Section 3 introduces our novel datasets, while proposed methods for MOT as well as age and gender estimation are discussed in Section 4. Experimental results of our methods are presented in Section 5, followed by a discussion of in Section 6. Finally, we conclude with Section 7.

## 2. Related Works

### 2.1. Multi-Object Tracking

A taxonomy of methods that solve multi-object tracking involve the way an MOT system processes video sequences, namely, online and offline. Online methods operate on the video in a frame-by-frame basis, and thus, perform tracking by only using information up to the current frame, a design principle that makes them suitable for trending applications. In contrast, offline methods have access to the entire video sequence, including future frames, as they process videos in batches. While the latter are designed to handle the problem of data association more efficiently by utilizing future information, they are limited in their applicability in scenarios with real-time requirements.

A different way to separate methods is the strategy that they follow to handle the different aspects of the MOT task. Most approaches to the problem of multi-object tracking (MOT) generally follow the *tracking-by-detection* design framework. They formulate the tracking problem as a two-stage workflow. A detection step which localizes targets in an image and a second data association step, where the goal is to match the detections with existing, corresponding trajectories, generate new ones in case a new target appears on the scene, or discard old ones, when a target is no longer visible. The second step in this paradigm involves the actual tracking part and typically consists of several subtasks, such as motion forecasting, embedding extraction (image representation), and data association, among others.

Most approaches that follow this strategy initially utilized two separate models, usually deep-learning-based architectures for their success in both tasks of detection and feature extraction. A popular choice is convolutional neural networks (CNNs) [32,33,34] even though, more recently, graph neural networks [35,36] and transformers [37,38,39] have also been used. The descriptive capabilities of deep networks have enabled these methods to achieve remarkable results, by continuously improving upon one of the two models, as they are both important in the final tracking performance. However, using two computationally intensive models entails some drawbacks. Most importantly, the computational overhead required to run both models prohibits their application in real-time scenarios because of slow running speeds. In addition, considering the fact that two resource intensive types of neural networks are typically used in both steps, this requires two separate training processes and also results in a significant amount of redundant computations that are generally similar and can be avoided.

To tackle some of these shortcomings, a similar approach has emerged that utilizes a single model to perform both steps of detection and target tracking, avoiding some of the aforementioned issues. Such methods jointly train a unified network to handle both tasks and are known as *joint-detection-and-tracking* methods [9,40,41,42]. Apart from applications in MOT, this design has also been applied to human pose estimation [43]. Similarly to the previous strategy, CNNs remain the most prevalent models for this task due to their significant research improvements over the last few years that enable them to handle both steps while constantly improving their accuracy and running speeds.

More recently, target association methods that rely solely on detector outputs to associate all detected bounding boxes have been proposed [44,45]. These methods match new detections with existing tracklets without the necessity of an embedding extraction step, which was typically handled by a deep learning network (e.g., [33]). Consequently, the use of a single computationally intensive module in the overall pipeline leads to a reduction in the system’s required resources and latency, rendering such methods accurate trackers with real-time capabilities, depending on detector performance. This design benefits from a simplified training procedure as well, since the only trainable component is the detector unit, as without an embedding extraction network, a second dataset is no longer necessary, reducing the amount of training data and enabling faster deployment.

Our work follows the strategy of online tracking-by-detection, where we employ a powerful object detector [46] to extract candidate targets in the scene readily available to the tracking component [44] for accurate consumer tracking in real-time retail video sequences while producing meaningful tracking outputs.

### 2.2. Age Estimation

The task of human age estimation has been well studied for a few decades by researchers. Age estimation techniques are often based on shape- and texture-based cues from faces, which are then followed by traditional classification or regression methods. Earlier approaches to the task utilized classic computer vision methods for feature extraction, such as Gabor filters [47,48], histogram of oriented gradients (HoG) [49], or local binary patterns (LBP) [50,51].

Currently, with advances in machine learning research as well as hardware capabilities, the predominant approach is the application of deep learning methods to solve the problem of feature extraction. CNNs have been widely adopted for their performance as capable feature extractors to obtain powerful representations of the input data. For instance, the works presented in [23,52,53,54] utilized convolutional-based networks and structures, whereas, Pei and co-workers [55] proposed an end-to-end architecture that uses CNNs as well as recurrent neural networks (RNNs). Duan et al. [56] combined a CNN with an extreme learning machine (ELM) [57], which is a feed-forward neural network that achieves very fast training speeds and can outperform SVMs in many applications, while the authors of [58,59,60] explored more compact and low resource convolutional models. Other deep learning methods, such as auto-encoders [23] and random forests [61], have also been adopted.

Most of these works make use of face images due to the fact that they provide more descriptive information about age ranges, since as people get older, certain common changes in facial characteristics can be observed, leading to better representations and higher accuracy of age estimation. Additionally, the majority of available corpora in the literature comprise media that depict faces exclusively, or at least contain face images, which are utilized after a detection and cropping step, discarding any other information.

Using images of the full body for this task has largely been an unexplored research topic, in part because of challenges in associating visual information from the body with apparent age, but also due to the lack of large publicly available datasets. Consequently, very few works have been proposed that use whole body images to estimate just the age of a person, for example, earlier approaches include [51,62,63], in which hand-crafted features were used. More recently, CNNs have been applied to the problem [53] obtaining accurate results demonstrating that full body images provide adequate visual information and can be successfully used to deal with this problem.

A subcategory to this problem is *apparent* age estimation, meaning that the actual age of the persons is not known beforehand, but is based on the subjective estimations of the annotator(s). In these methods, evaluation is performed on apparent ground-truth data [64]. Due to the nature of real-world data, apparent age estimation is a well-suited subclass for real-time applications where visual perception of age plays an important role. To the best of our knowledge, our work is the first to tackle this problem using only images of the full body as input, instead of the usual datasets of faces.

### 2.3. Gender Classification

The task of classifying the gender of people that appear in images is similar in nature with that of estimating their age. Over the last decades, a few works that focus solely on this task have been proposed. Conventional methods rely on shallow-learned features, such as histogram of gradients [65,66] or local binary patterns [67,68,69] for feature extraction and support vector machines for classification [30,70] and still remain popular and are widely used.

As with most image processing and computer vision problems, CNNs have also been adopted for gender classification, usually to obtain robust representations [71]. For example, Aslam and colleagues [72] propose wavelet-based convolutional neural networks for gender classification, while isolated facial features and foggy faces are used as inputs in CNNs in [73,74]. Ref. [75] provides a comparison of traditional and deep-learned features for gender recognition from faces in the wild, and [76] explored several popular convolutional architectures used in other tasks for identifying the gender of humans wearing masks.

Since images of the face contain more relevant information about gender compared to full body, they lead to better accuracy, and therefore, most methods that have been proposed for this task utilize datasets that contain images of faces. This reason is also an additional factor that contributes to the lack of publicly available full-body datasets. In contrast to age estimation, using full body images for this task has received some attention [30,65,66], but still remains an open area of research. Some methods deviate from the standard approach of using two-dimensional images to the application of three-dimensional data for gender recognition to alleviate some difficulties present in 2D data [77,78].

A different avenue of research for this problem is the combination of different modalities to assist with performance by taking advantage of features from different sources. More specifically, multi-modal data, such as depth [79] or thermal images [31,80,81], of the body have also been explored as auxiliary inputs to classification systems for improving performance and helping to overcome challenges arising when only RGB images of the body are available.

Apart from aforementioned approaches, a few works have focused on gait [82,83,84] as an indicator of gender. Gait-based methods assume information accrued from the gait of a person, which is related to change of pose in consecutive frames. The typical assumption is a controlled environment where multiple views of the objects are available so that the change in pose can be determined [85]. As a consequence, this limitation does not allow gait-based methods to be employed for practical consumer demographic estimation.

### 2.4. Related Age and Gender Multi-Attribute Classification Methods

Both the age and gender information about a person can be estimated from face images with great accuracy, and therefore, several works have been published that attempt to solve both tasks. Due to challenges present when using body images as previously discussed, as well as owing to dataset availability, the preferred form of data used by these works favors facial images. One of the earliest methods can be found in [86,87], where classic image processing techniques are employed to extract information based on textures of wrinkles and colors. More recently, Eidinger et al. [88] proposed a SVM-based approach for age and gender classification from face images in the wild.

With advances in deep learning, various CNNs have been adopted for predicting age along with gender, replacing older methods, typically comprising feature extractors as parts of larger systems or end-to-end models that handle the additional process of classification. For example, all works presented in [89,90,91,92,93,94] used only convolution-based architectures to tackle both problems with images of faces as inputs, whereas Uricár and co-workers [95] proposed a combined CNN feature extractor with a SVM classifier. In a similar fashion, Duan et al. [96] developed a hybrid technique that utilizes CNNs for feature extraction, whereas classification is handled by an extreme learning machine (ELM) for faster training and more accurate predictions. Another hybrid method that leverages non-convolutional neural networks and CNNs by fusing their decisions for a final prediction is presented in [97]. Lately, owing to their success in various tasks, vision transformers have also been explored for age and gender classification [98].

Using full body images is a much more rare approach, and in this case, most works that classify age as well as gender do so as part of a multi-attribute classification problem, where the goal is to predict a larger set of attributes. Analogous to the problem of gender-only estimation, gait-based methods have also been developed for the combined task, featuring multiple views of a person’s entire body [99], operating on a single image in real-time [100], or employing data from wearable sensors [101]. However, such approaches often assume a controlled monitoring environment of the involved subjects of interest, not readily applicable in real-time consumer tracking.

## 3. Materials and Dataset Preparation

In this study, two large datasets are introduced which can be made available upon email request to the corresponding author. Furthermore, data are uploaded in a privacy-protected (anonymized) format wherein facial parts are occluded on purpose for compliance with data privacy regulations. In this respect, we distinguish two types of data sources. The first type comprises facial blurring of the images where faces are present using deface (see Section 1.3) software. The latter type is based on deep-anonymity software (see Section 1.3) that replaces actual faces with artificially generated ones.

### 3.1. Data Acquisition

To record videos for the dataset, cameras are installed in retail consumer stores in key positions overlooking the main isles or most of the store where possible. The installation locations were selected after taking into account the store’s architecture and the cameras’ view areas, with the intent to capture as much movement of consumers as possible. An additional requirement is to retain visibility of the shelves and in turn their products in order to extract meaningful associations between consumers and their interactions with the shelves (e.g., total standing time in front of the shelves, potential purchase, general preference), their path inside the store, or even achieve the end goal of estimating their preferences. In total, four cameras are used for one retail store: three conventional RGB cameras and one thermal. The latter is placed in close proximity with one of the RGB cameras and physically aligned, in a way so as to depict as much of the same region as possible as its pair due to differences in their inherent intrinsic parameters (focal length, zoom, aperture, field of view, etc.).

The cameras use sensors that are motion triggered, which allows for video recording only when motion is detected and thus control the volume of captured videos so as to avoid redundant data, especially on busy days where traffic is high. Furthermore, the recordings can be activated by peripheral movement, i.e., a consumer that briefly appears in the periphery of the field of view without being fully visible in the frame. As a result, a significant amount of captured videos contain either a small number of consumer occurrences or none at all (partial appearances). In order to collect videos with appropriate amounts of movement and interactions between consumers and products, a simple heuristic method to discard unwanted videos was devised. First, an automated script dynamically parses each recorded video by applying a vanilla object detector [102] to obtain consumer detections in a frame-by-frame basis. Then, the pruning rule determining which video will be kept for our dataset was based on the average number of detections being higher than a user-defined threshold (e.g., two or more instances are enough to avoid video pruning).

The previously described heuristic achieves the goal of filtering videos according to their content in terms of consumer appearances. After this filtering step, a different script parses the remaining videos and creates the resulting dataset for the annotation platform (see Section 3.2). By default, the cameras capture videos at 30 FPS and since consumers in retail stores tend to move slower compared to outdoor scenes, the frames in the resulting videos are visually similar with minor movement changes due to the sample rate, resulting in an excessive amount of redundant frames that make annotation a cost-prohibitive process with large overlaps of duplicate ground truth data. For this reason, we apply subsampling to the original video data by dropping frames without loss of visual coherence in the final video. In other words, we prune 20 frames per second, thus yielding approximately 10 frames per second in the subsampled output sequence.

In total, after filtering, 145 video sequences were collected from RGB cameras in indoor areas of the retail store, 25 of which are used for testing. The same split (120–25) is followed for both datasets. The videos feature sufficient illumination conditions, with multiple consumers per frame exhibiting regular or irregular movement patterns along with potential occlusions due to consumer interactions or their position with respect to the shelves. In contrast with existing MOT datasets (e.g., MOT16 [103]), the scale of targets is generally larger, whereas the cameras are static in all sequences. Additionally, a challenge of this dataset is the presence of reflective surfaces (e.g., glass panels) that can produce false positive detections. The frame resolution is 3072×1728 and the average length of the videos is 60 frames, yielding a total of approximately 8700 images. Example images showcasing challenges of the *Consumers* dataset are illustrated in Figure 1.

### 3.2. Data Annotation

Towards the direction of obtaining training data for our algorithms, we have developed an annotation platform in Java Spring Boot (https://spring.io/projects/spring-boot accessed on 26 October 2023) which comprises an open-source framework for scalable applications that provides an interactive interface. Therein, a registered user can log in using their credentials, then select a video from a list of videos that are uploaded to the application so as to start the annotation task, namely, the creation of ground-truth bounding-box detections and age/gender estimates for each detected instance.

**Figure 2:**
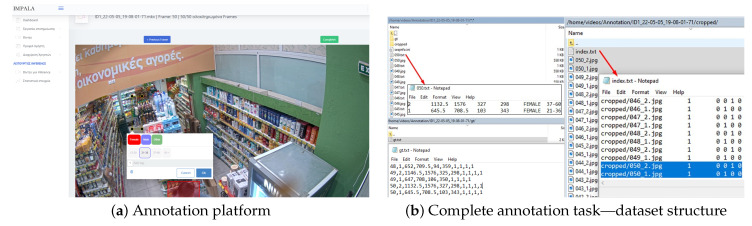
Overview of the *Consumers* and *BID* datasets’ creation using the annotation platform developed for the paper’s needs. (**a**) Annotation platform (**b**) Left: File structure of the *Consumers* dataset for a given sequence (top) and its corresponding ground truth (bottom). The part on the right refers to a subset of the *BID* dataset that is created during the annotation process and consists of cropped images from that sequence and their respective ground truth representations as vectors.

For each frame of a video sequence, the user actually marks the location of bounding boxes of potential targets by clicking on the image and surrounding the target boundaries. Subsequently, the user assigns an ID to each target with a convention followed by all annotator users, i.e., from top-left to bottom-right of the image. This facilitates the consistency when assessing tracklet association between different IDs during MOT performance evaluation. Moreover, the annotator provides a subjective estimate of the targets gender and age range. Then, the user proceeds to the next frame and repeats this process for all frames until the end of the video sequence. When a single frame is entirely processed, a cropping algorithm creates the appropriate body images of each annotated target into a separate file structure particularly intended for the body image dataset. We should note here that anonymization takes place right after an annotation task is complete (i.e., the complete button also triggers the deface algorithm). Upon completion of the annotation procedure, the platform creates all the necessary files containing the ground-truth coordinates and demographic information of each bounding box for each frame along with corresponding, anonymized images. Figure 2 summarizes the annotation pipeline.

Since the procedure to annotate all frames in a sequence can be tedious and time-consuming, the platform creates files on a frame-by-frame basis, effectively allowing users to pause their work and resume it later from the same frame, or to examine previous frames and make corrections in cases of wrong or missing ID assignments. The system keeps track of all annotation jobs and displays this information to the user in terms of completion percentage so that no frames are missed.

### 3.3. Consumers Dataset

**Figure 3:**
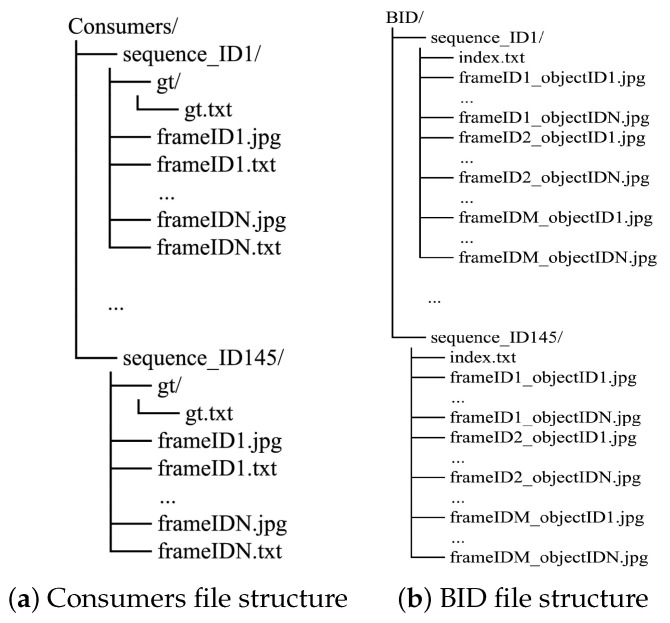
Dataset file stucture of *Consumers* (**a**) and *BID* (**b**).

In line with the previous section, from the 145 selected sequences in total, 120 form the training set and the remaining 25 are used as the testing set. To comply with privacy-respecting regulations, all frames that contain facial information are anonymized by blurring the area of the face or replacing it with an artificially generated one. In cases wherein a person’s face is missed (not detected) by the automated anonymization software, we manually perform the blurring, to ensure that all frames are anonymized. In Figure 3, we overview the datasets’ file structure. *Consumers* is organized as follows: video sequences are arranged in separate folders each folder corresponding to their sequence IDs. Each sequence is split into distinct image frames with ascending names, and each image file is paired with a corresponding text file containing ground truth information about object instances, if they exist (no detections correspond to empty ground truth text files). Annotations are provided in the form of consumer/target ID number, bounding box coordinates (center point, box width, and height), as well as demographic information about the consumer (age and gender group) per annotated instance in the frame (see Figure 2b-left), with the same consumer retaining their identity for as long as they appear in the video sequence. In cases where the same identity exits and re-enters the scene, the same ID number is used. Apart from the per-frame annotations, which can be used to train object detectors, a text file “gt.txt” containing the ground truth for the entire sequence is provided in a subfolder. This file is formatted in the same way as the MOT benchmarks (e.g., MOT17), and is used for the evaluation of tracking performance (Section 5).

### 3.4. BID (Body Image Dataset)

**Figure 4:**
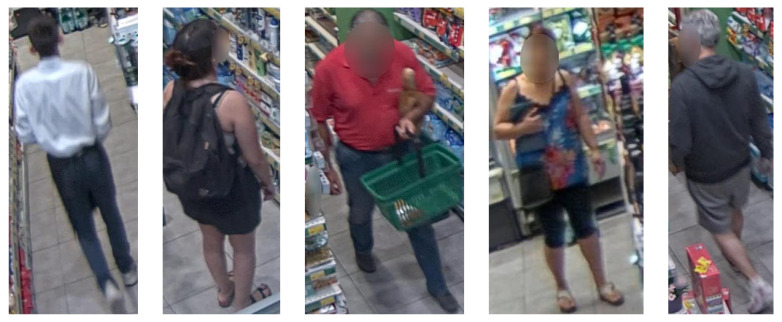
Sample images from the *BID* dataset showcasing instances of consumers from both genders and various age groups.

Using the provided annotations from the annotation platform, croppings of the original image are created where each cropping corresponds to a single instance of a target consumer, which is also reflected by the image name (frameID_consumerID, see Figure 2b on the right). Although the dataset’s intended purpose is apparent demographic estimation, this information can be also utilized for other computer vision tasks, such as person re-identification. The cropped images contain the full body of the consumer and any facial information is expunged as previously. The dataset (see Figure 4, where some examples are illustrated) features a large diversity of consumer appearances due to clothing and wearable accessories that create ambiguities with respect to the ground truth of the data (*apparent* estimation). Consumers of several age groups are also present, along with permutations of gender and age group interval. Four age groups that offer a general representation of consumers in stores were selected: below 12–20 years, 21–36, 37–60, and over 61.

Due to the fact that some frames contain zero instances of consumers, such as the beginning of a sequence, and multiple instances in some frames, the overall number of images in this dataset is 6641 with various resolutions and aspect ratios, enabling the training of models with different input requirements. The file structure is similar to *Consumers*, where each sequence uses its own directory and a single text file is provided with ground truth information about all images in the current directory (see Figure 3b). Annotations are provided in the form of binary vectors for each target’s apparent gender and age group in the form: [GENDER  AGE_GROUP_1  AGE_GROUP_2  AGE_GROUP_3  AGE_GROUP_4], where GENDER denotes the target’s gender (1 for female, 0 for male), and the following four values are a 1-hot representation of the ascending age group. For example, a ground truth representation of [1 0 1 0 0] denotes a consumer instance of a female in the 21 to 36 age group.

## 4. Proposed Methods

### 4.1. Multi-Consumer Visual Detection and Tracking

The problem of multi-object tracking can be formulated as follows: given a sequence of *N* image frames with *K* total targets, the goal of a tracker is to output a set of trajectories $\hat{T}=\left\{{\hat{t}}_{1},\cdots ,{\hat{t}}_{K}\right\}$ for each target in that sequence that is as close to the ground truth trajectories $T=\{t_{1},\cdots ,t_{K}\}$ as possible. Each trajectory is represented by a series of bounding box locations $B=\left\{b_{1},\cdots ,b_{N}\right\},b_{i}\in R^{4}$, for that target over the length of the sequence. Unless provided, the bounding boxes need to be detected using any method of target detection.

Our work follows the tracking-by-detection approach, utilizing a powerful detection network [46] to localize targets and corresponding detection scores in a source image, which are then fed to a robust association strategy (ByteTrack [44]). Following our previous work [104], we add an LSTM module for motion forecasting, replacing the conventional Kalman filters. The resulting pipeline is simple, yet powerful, yielding acceptable running speeds without compromising tracking performance. By reducing the amount of parameters in the detection network, accuracy can be slightly lowered in favor of latency, allowing for deployment in various real-world scenarios depending on the available hardware capabilities.

#### 4.1.1. Target Detection

We employ YOLOX [46] as our base target detection network. YOLOX is a family of robust and efficient object detectors that builds upon earlier YOLO methods and utilizes advanced practices from the literature to achieve better performance in terms of detection accuracy as well as computational requirements. In this subsection, we briefly summarize some of the key improvements of this detector that enable it to achieve better results compared to its predecessors.

**Anchor-free design**. In contrast to anchor-based earlier YOLO methods (e.g., YOLOv4 [105]), YOLOX models switch the detection head to an anchor-free design in order to alleviate some limitations of the anchor mechanism, such as the increased complexity of the detection heads due to multiple anchor points that also need to be computed before training. Moving to an anchor-free design simplifies the architecture of the detector reducing the amount of parameters required, and therefore, speeding up training and inference. Even though complexity is reduced, performance remains on par with anchor-based detectors.

**Figure 5:**
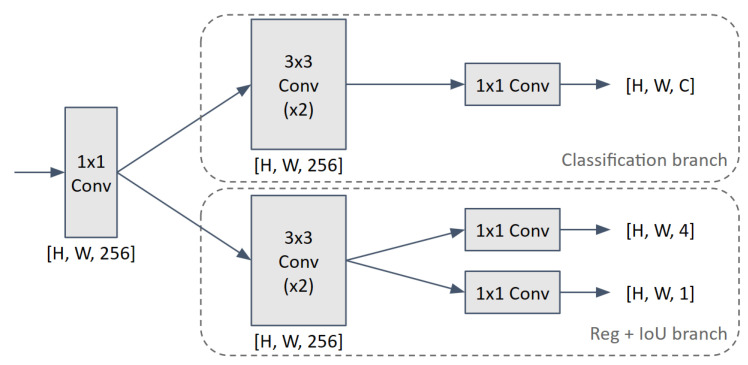
The lightweight decoupled head used in YOLOX. While traditional methods use a single branch for both tasks of detection and classification, this formulation uses two separate branches to avoid potential conflicts. The head is added at different feature levels in the backbone network.

**Decoupled detection head**. Another significant feature of this model family concerns decoupled architectures, which replace standard coupled detection heads. The purpose of this design choice is to reduce the conflict between the tasks of regression and classification which is present in other detectors, such as the YOLO series, that add coupled detection heads for both tasks at different feature levels. The decoupled head used in YOLOX has two separate parallel convolutional branches, one for each task. Benefits of the decoupled heads include an increased convergence speed and improved performance by simplifying the training process. A 1×1 convolution is used to reduce the feature dimensions, followed by two distinct branches, each with two 3×3 convolution layers. The lightweight head used in YOLOX is depicted in Figure 5.

Furthermore, the authors adopt a simplified method for label assignment that is based on OTA [106], dubbed *SimOTA*. This method treats the label assignment procedure in object detection as an Optimal Transport problem and simplifies the process by approximating the solution using a top-k strategy. The simplified assignment strategy reduces training time while at the same time increasing average precision (AP). For an in-depth analysis of *SimOTA*, as well as other innovations involved in YOLOX, we refer the reader to [46].

**Figure 6:**
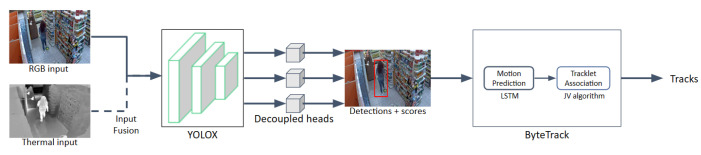
Proposed system’s pipeline for multi-object tracking with optional thermal modality. The system’s input is a sequence of images (or pairs). Detections and their corresponding scores are produced by the detection network and fed to ByteTrack for association to output the final tracks for the sequence.

Our proposed pipeline is capable of utilizing an additional thermal input modality when it is available. In this case, two sequences of the same length are used as inputs and are processed frame-by-frame in parallel. Each frame pair is linearly combined at the input stage (with the RGB component being more prevalent) and the result is fed to the detector network. A prerequisite of this function is a preprocessing step performed in one or both sequences so that they are trimmed to the same length (or number of frames) and are visually aligned to depict the same area of the store. This form of early fusion can help prevent visual noise in scenes with reflective surfaces. We should note here that our approach to multi-modal input fusion is rather straightforward, since both RGB and thermal modalities are typically linearly combined, as opposed to more refined and structured data fusion methods which actively learn a common subspace [107,108,109], wherein distinct modalities, or multiple views of the same modality, can be aggregated into discriminative representations. Nonetheless, this is not a hindrance for our problem, since we observed that thermal modalities did not actually contribute much to the accuracy of the MOT task, and thus, we did not investigate further possible subspace learning techniques. The entire pipeline is illustrated in Figure 6.

#### 4.1.2. Target Tracking

The next step in the visual tracking pipeline for the detected targets accrued from the previous phase involves the *motion prediction* part. Therein, our key approach for the proposed tracker replaces Kalman filters with an LSTM network able to model a sequence of consecutive t−k past frames up to frame t−1 to predict bounding-box coordinates for each detected target between the current (*t* frame) and previous (t−1) frame. This in turn increases tracking performance contrary to Kalman filters that model detected targets only between the previous and the current frame.

For a given sequence, the tracker maintains information about tracks in the form of lists. Depending on their tracking state, tracks can be active (tracked) or lost. If, in the current frame, a detection has been assigned to a track that was previously stored, regardless of its state, it is considered active for the current frame. Tracks that were previously active but are not assigned to a detection in the current frame are marked as lost.

The assignment procedure of ByteTrack works as follows: after a frame has been processed by the detector along with non-max suppression, final detections in the form of bounding box coordinates and their corresponding detection scores are obtained. Bounding boxes are separated into two categories depending on their detection score: high score boxes, if their score is greater than a predetermined *tracking threshold*, and low score ones, if that score is lower. Motion prediction is applied to all previously active tracks aiming to estimate their locations in the current frame. An additional role of motion prediction is to act as a constraint on the plausibility of associations, rejecting matches between detections and tracks that are spatially far away.

Following the motion prediction operation, the next step of the tracking pipeline comprises *tracklet association*, wherein a first association takes place by assigning high score boxes with previous tracks. Then, a second association between the remaining tracks and lower score bounding boxes occurs. These assignments determine the tracking status of all tracks:

- For tracks with assigned detections in the previous frame that are matched, they retain their status as active.
- If a match is found for tracks that have had no detections assigned to them for some frames (lost), they are now considered as active.
- The remaining tracks after both association operations are marked as lost, since no matches were made for them in the current frame.
- After these previous checks, all remaining detections initialize new active tracks.

Finally, tracks that have been marked as lost for a number of frames that exceeds a pruning threshold are removed.

In both association operations, IoU distance is used as a measure to determine the distance of tracks and detections, and the linear assignment problem is solved using the Jonker and Volgenant algorithm [110].

### 4.2. Apparent Age and Gender Estimation or Recognition

The goal of apparent age and gender estimation from full body images is to predict these attributes with as much accuracy as possible in cases where facial information is not available. In the absence of face images, only information from the full body can be exploited, increasing the estimation process difficulty, as discussed in Section 1.2, Section 2.2 and Section 2.3. In real-world scenarios, such as in retail stores, security cameras are usually used to capture the movements of consumers while subjecting to privacy respecting laws, resulting in produced data where the face is unavailable, e.g., blurred video or low resolution. For applications in such an environment, using cues from the full body is the only avenue to tackle the problem and therefore a system utilizing full body images as its main source of data requires a high degree of robustness.

In this section, we discuss the convolutional network architecture, which is also integrated in our end-to-end system for apparent demographic attribute estimation. The employed network enables the joint learning of age and gender cues by predicting a holistic representation for both attributes, without requiring task-specific network architectures, thereby allowing practical deployment in real-time applications.

#### Multi-Attribute Classification

To estimate the consumers’ apparent age and gender, we use a simple framework consisting of a feature-pyramid-based backbone network enhanced with additional attribute localization modules [111] to accurately predict the regions in the image that contribute to each attribute. The architecture of the network is based on *Inception* [112], which exploits the pyramid-based structure to take advantage of features from different depths of layers from the network that provide complementary information.

**Figure 7:**
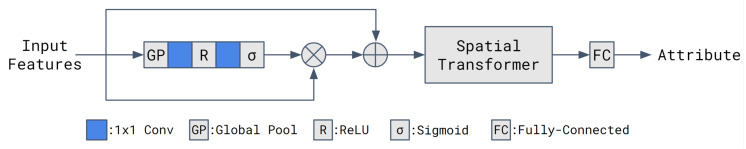
Depiction of an attribute localization module [111]. The input is a combined feature vector and the output is a prediction for a single attribute. This module is used in this work.

At the end of multiple inception blocks where features are accumulated from different pyramid levels (corresponding to different scales), localization modules are added. By combining information from different scales, the model can improve its ability to recognize attributes at various sizes and locations within the image. The localization modules attempt to detect regions in the image that are responsible for each attribute. These regions are represented internally as bounding boxes. The architecture of these modules consists of a simple channel attention network [113] (consisting of global pooling and convolutional and ReLU layers with sigmoid activation), a spatial transformer [114], simplified to apply scaling and translation operations to express the bounding box, and a residual connection to preserve information from the input. The spatial transformer treats regions in the image that are responsible for each attribute as simple bounding boxes that can be calculated using a combination of scaling and translation operations as follows:

(1) $$\left(\begin{array}{c} x_{i}^{s}\\ y_{i}^{s} \end{array}\right)=\left[\begin{array}{ccc} s_{x} & 0 & t_{x}\\ 0 & s_{y} & t_{y} \end{array}\right]\left(\begin{array}{c} x_{i}^{t}\\ y_{i}^{t}\\ 1 \end{array}\right),$$

where $t_{x},t_{y}$ and $s_{x},s_{y}$ are, respectively, the translation and scaling parameters that can be used to calculate the bounding box. In Equation (1), $\left(x_{i}^{s},y_{i}^{s}\right)$ correspond to the source coordinates for the *i*-th pixel, and $\left(x_{i}^{t},y_{i}^{t}\right)$ to the target ones. The values of $t_{x},t_{y}$ are constrained to the (−1,1) range to reduce convergence time. Similarly, and for the same reason, $s_{x},s_{y}$ are limited to (0,1). Each module outputs a prediction for a single attribute. Figure 7 shows the structure of a localization module.

Training takes advantage of the deep supervision method [115], where ground-truth data are used as direct supervision for each attribute prediction output from the branches of the network. A voting scheme is added to leverage the different predictions that are generated at each feature pyramid level, selecting the best for each attribute region. Weighted binary cross-entropy is selected as the loss function for each branch of the network (the forward branch and three groups of localization modules that form the second branch):

(2) $$L_{total}=L_{fwd}+\sum_{l=1}^{3}L_{l},l\in 1,2,3.$$

(3) $$L_{l}\left({\hat{y}}_{l},y\right)=-\frac{1}{N}\sum_{n=1}^{N}\gamma^{n}\left(y^{n}log\left(\sigma \left({\hat{y}}_{l}^{n}\right)\right)+\left(1-y^{n}\right)log\left(1-\sigma \left({\hat{y}}_{l}^{n}\right)\right)\right)$$

In the above equations, $L_{fwd}$ indicates the network’s forward branch loss, whereas $L_{l}$ indicates the loss for each localization module group, *N* refers to the number of attributes, which is dependent on the dataset (for the *BID* dataset N=5), σ is the sigmoid activation function, $\gamma^{n}=exp\left(-\alpha_{n}\right)$ is the loss weight for attribute *n*, $\alpha_{n}$ is its prior class distribution, and $y^{n}$ and ${\hat{y}}^{n}$ refer to the ground truth and the network prediction for attribute *n*, respectively. The total loss is calculated by summing the forward branch loss as well as the loss for each group of localization modules.

**Figure 8:**
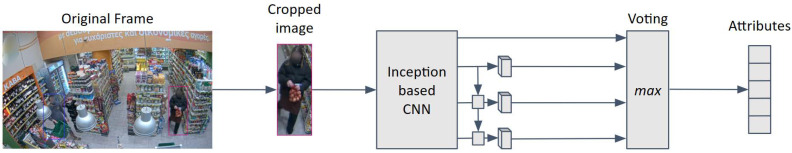
The multi-attribute classification network used in our work. The input is a cropped image of a single consumer that can be either produced beforehand by data pre-processing or in real-time from the output of the detector. The four prediction vectors from the architecture are used in maximum voting to determine the final attribute predictions which is a binary N×1 vector for *N* attributes.

Owing to its relatively simple architecture and low computational cost, the attribute classification pipeline achieves fast running speeds with acceptable accuracy, and as a result, can be integrated in the tracking network to output demographic predictions for the tracked targets in real-time in addition to the bounding boxes without significantly harming performance. The multi-attribute classification network is illustrated in Figure 8.

## 5. Experimental Results

### 5.1. Datasets and Evaluation Metrics

#### 5.1.1. Visual Tracking Evaluation Protocol

We evaluate our proposed tracking framework on our indoor *Consumers* dataset. We use six different models from the YOLOX family as detectors for our experiments. Their size in millions of trainable parameters and computation costs are reported on Table 1. Weights are initialized by the procedure described in ByteTrack [44] in which a mixed dataset containing images from the training sets of MOT17 [103], CrowdHuman [116], ETHZ [117], and Citypersons [118] datasets is created and used to train the detectors when the objective is testing on the MOT17 test set.

The MOT16/17 datasets contain the same 14 video sequences, 7 of which are used in the training set and 7 in the testing set, with the difference that MOT17 provides three sets of public detections for each sequence in the training set improving annotations by increasing the bounding box accuracy and adding missing detections. Various conditions are present among scenes, such as several annotation classes or different ambient lighting depending on the time of day. Large variations in crowd sizes as well as target scales are also found. An additional challenging factor is related to the recording cameras used for capturing the scenes, some of them are moving while some are static. Even though most sequences are from outdoor cameras, there exist a few that depict indoor scenes. The total number of frames for training amounts to 5316. For each training sequence, its first half is used in the mixed dataset for training, and the remaining half for validation.

The ETH dataset contains 1804 images in video clips recorded from a camera mounted on a car or a chariot. Bounding box annotations and scores of pedestrians are provided. Scenes that overlap with the MOT17 test set are removed from the mixed dataset, following the literature assumptions.

CrowdHuman (CH) is a large, richly annotated benchmark dataset for evaluating detectors in crowd scenarios. It contains 15,000, 4370, and 5000 images for training, validation, and testing, respectively, offering a high degree of target variation. There are a total of 470,000 human instances from train and validation subsets and 23 persons per image, with various kinds of occlusions. Each human instance is annotated with a head bounding-box, human visible-region bounding-box, and human full-body bounding-box.

CityPersons (CP) is a subset of the larger Cityscapes dataset that focuses on pedestrian detection. Bounding box annotations are provided for pedestrians, including riders and sitting persons. The training subset features various weather and illumination conditions, with an average density of 7 persons per image. There are 2975 images in the training set with almost 20,000 unique persons.

We employ ByteTrack’s pre-trained weights for all detector networks and fine-tune for 20 epochs on the raw train set of our *Consumers* dataset as we found that the initialization obtained by training on the mixed (MOT-CH-ETH-CP) dataset provides a strong baseline for target detection that is robust with regard to different scales and appearance scenarios. The image training size for YOLOX-X, YOLOX-L, and YOLOX-M is set to 800×1440, while for the remaining models, it is lowered to 608×1088. All other training settings and augmentation protocols remain unchanged, as in [44]. We note that while fine-tuning on the *Consumers* dataset is performed using the raw training set data, the evaluation in our experiments uses the anonymized test set. For reproduction of results, we release the final weights for all models (Code is available at https://github.com/jpanagos/consumers-bid).

Considering the motion forecasting module, in order to ascertain the effectiveness of the LSTM network in tracking performance, several configurations with varying amounts of neurons and layers were trained, and the best performing setup was selected. The training procedure of [119] was followed, which employs the Adam optimizer with a starting learning rate of 0.001 that is multiplied by 0.1 at the 60th epoch. The LSTM networks are trained on the MOT17 dataset for a maximum of 100 epochs. An ablation analysis of the effect of the LSTM network in overall MOT performance is included in the Appendix A.2.

#### 5.1.2. Performance Indices

We evaluate the models based on the standard metrics used by the literature for assessing tracking performance, which are known as the *CLEAR* MOT metrics [120]. These include MOTA, MOTP, and IDF1.

MOTA refers to Multi-Object Tracking Accuracy and is one of the most commonly referred metrics by MOT works. It is defined as:

(4) $$MOTA=1-\frac{\sum_{t}\left(FN_{t}+FP_{t}+IDS_{t}\right)}{\sum_{t}GT_{t}},$$

where *t* is the frame index and GT is the amount of ground truth objects and FN, FP, and IDS denote the false negatives, false positives, and identity switches, respectively. A target missed by any hypothesis is considered a FN, while a target that is wrongly hypothesized is a FP. An identity switch occurs when a target is given a different ID in the next frame. True and false positive detections are determined with an IoU overlap percentage greater than 50% between the predicted bounding box and its corresponding ground truth. MOTA is expressed as a percentage but it can also be negative when a tracker makes errors that exceed the number of actual objects in a scene.

Multi-Object Tracking Precision (MOTP) is another index that is regularly used in the literature. It measures the localization accuracy of the detector in terms of the dissimilarity between all true positives and their corresponding ground truth targets:

(5) $$MOTP=\frac{\sum_{t,i}d_{t,i}}{\sum_{t}c_{t}},$$

where $c_{t}$ denotes the number of matches in frame *t* and $d_{t,i}$ is the bounding box overlap of target *i* with its assigned ground truth object.

Additionally, we use the identity F1 score (IDF1). IDF1 is the ratio of correctly identified detections over the average number of ground-truth and computed detections. This metric focuses more on identity matching ability and data association performance:

(6) $$IDF_{1}=\frac{2\times IDTP}{2\times IDTP+IDFP+IDFN},$$

where IDTP, IDFP, and IDFN represent the true positive ID, false positive ID, and false negative ID measures, respectively.

For each track within a sequence, the mostly tracked (MT), partially tracked (PT), and mostly lost (ML) metrics measure the quality of tracking. Each target is assessed using these metrics according to a tracking percentage:

- Mostly tracked (MT): if it was successfully tracked by the algorithm for more than 80% of its trajectory;
- Mostly lost (ML): if less than 20% of its trajectory is tracked correctly;
- Partially tracked (PT): otherwise.

Ideally, a tracker should achieve high MOTA, MOTP, and IDF1, and most of its targets should be classified as mostly tracked, while mostly lost targets should be as few as possible.

As an indicator of latency, we report the total running time of the entire model, which includes the time required for detection network inference and data association, expressed as frames per second (FPS). To that end, all tracking experiments are repeated five times and reported FPS values are averaged. This metric is useful for application deployment in real-time scenarios where speed is a requirement. Furthermore, it allows us to measure the impact of the LSTM’s network additional computations on the overall speed of the system.

#### 5.1.3. Age–Gender Recognition Evaluation Protocol

The backbone network of the age and gender prediction model is initialized using ImageNet pre-trained weights and (whole network) fine-tuning is performed on the raw *BID* training set for a maximum of 100 epochs. An initial learning rate of 0.0001 is used and multiplied by a factor of 0.1 at the 50th epoch. A batch size of 32 is used along with the Adam optimizer at default settings and a weight decay of 0.0005.

After training, we evaluate the model on the *BID* test set. Our goal is the accurate estimation of the apparent gender and age group of each target. We report the following measurements:

- Mean Accuracy (mA) = $\frac{1}{2N}\sum_{i=1}^{M}\left(\frac{TP_{i}}{P_{i}}+\frac{TN_{i}}{N_{i}}\right)$;
- Accuracy = $\frac{TP+TN}{TP+TN+FP+FN}$;
- Precision = $\frac{TP}{TP+FP}$;
- Recall = $\frac{TP}{TP+FN}$;
- F1 score = $\frac{2TP}{2TP+FP+FN}$,

where *N* indicates the amount of samples in the dataset, *M* is the number of attributes (M=5 in our case), $P_{i}$ and $N_{i}$ are the number of positive and negative examples for the ith attribute, and $TP_{i}$ and $TN_{i}$ are the correct respective predictions. TP and TN refer to the true positives and negatives, while FP and FN are the false positives and negatives, respectively. A retrieved result (classification vector) is considered as TP when both age and gender are correctly predicted.

### 5.2. Numerical Results

#### 5.2.1. Multi-Object Tracking

We evaluate our method (see Table 2 and Table 3) as well as several recent MOT trackers on our *Consumers* dataset after the anonymization process. These include SORT [32], DeepSORT [33], MOTDT [121], ByteTrack [44], OC-SORT [45], and StrongSORT [122]. A common feature of these trackers is the use of Kalman filters for motion forecasting, which allows for a comparison with our method for indoor consumer tracking. It should also be noted that some methods (e.g., DeepSORT), contrary to our approach, require an additional re-identification module in order to perform tracking. This typically comprises a CNN-based architecture trained on pedestrian re-identification datasets and introduces additional computation, which slows down their runtime.

#### 5.2.2. Apparent Age and Gender Estimation

We also evaluate the performance of several reference methods primarily developed for pedestrian attribute recognition [111,123,124,125] on our *BID* dataset. All methods are trained on our *BID* dataset using their default settings.

Experiments are performed using three data setups. In the first setup, we use the raw (non-private) data as they are obtained from the annotation platform for both training and testing in order to establish age and gender estimation baselines without any additional noise (e.g., face area blurring). Results for the aforementioned methods with different backbone networks are shown in Table 4.

In the second setup, two functionally distinct anonymization methods (see Section 3) are applied to the entire dataset and the final anonymized images are used for training and testing, allowing a thorough assessment of each method’s robustness to information loss that occurs when a section of the image is blurred. Table 5 includes the results for the deface-anonymized data (blurring of the face area).

To ensure that the method we use on our end-to-end system does not rely on any facial data, we evaluate all methods on the anonymized *BID* test set while using the weights obtained from training on the raw dataset. The results of this experiment are shown in Table 6. We believe this setup to be a more representative use case of the method in the industry which better reflects real-world conditions, where an application would potentially blur privacy-sensitive areas of the image before applying any form of estimation. Regarding the experimental setup with deep privacy anonymization (replacing the face with an artificially synthesized one), the reader is referred to Appendix B for a similar analysis of numerical results.

Finally, an ablative study is presented in Table 7, where we analyze the sensitivity of the attribute localization module to different attention methods and their parameters and its effect on the obtained accuracy. We experiment with two different lightweight attention methods, namely Squeeze-And-Excitation (SE) [113] and Shift-And-Balance (SB) [126]. Both methods introduce a subnetwork consisting of a global average pooling layer followed by a bottleneck of two fully-connected layers around a ReLU non-linearity function. The bottleneck shrinks and subsequently expands the channels back to the original amount, while the output of each attention method is then combined with the input via a residual connection. While the former (SE) models the channel-wise relationships by generating an attention mask which is then multiplied with the layer’s input, the latter introduces a learnable control factor, which scales the attention output before performing the addition operation, thus moderating the effect of the attention scores in the input, and thereby balancing the feature contributions from each component. This design allows the attention methods to first extract the most relevant information about the channels and then to model interactions and dependencies between the channels of the intermediate feature vectors.

Since a form of dimensionality reduction which controls information flow is involved within the bottleneck architectures of both methods, we perform experiments with regard to the hyperparameter affecting the amount of channel reduction to determine the ideal value that provides the best results for our *BID* dataset. More specifically, a reduction ratio decreases the channel dimensions of the output tensor after the first fully connected layer is applied. Therefore, manipulating this value regulates the amount of information loss that occurs at this layer.

#### 5.2.3. End-to-End Qualitative Results

**Figure 9:**
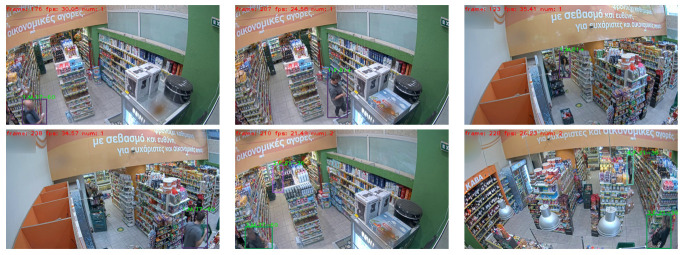
Qualitative examples (selected frames) of the end-to-end system when applied to out-of-dataset consumer video sequences, i.e., images not present in the *Consumers* dataset, but captured from the same cameras. Images showcase challenging intra-class variations concerning difference in age or gender, even when facial information is absent. Images are best viewed in color.

In this section, we present example qualitative results of the end-to-end system comprising the proposed consumer tracking approach combined with the selected demographic prediction method (Inception-based backbone [111]) on the detected instances, when tested on challenging out-of-dataset consumer video sequences (see Figure 9).

## 6. Discussion

From our experiments, as presented in Table 2 and Table 3, the use of the LSTM network for motion estimation provides a significant benefit for multi-object tracking, improving all tracking quality metrics, compared to the standard use of Kalman filter (KF). More specifically, higher improvements are observed for the MOTA and IDF1 scores, while MOTP improves as well, albeit at a lesser degree. Tracking quality also obtains benefits, with the amount of mostly tracked trajectories increasing, while simultaneously the partially tracked ones are reduced, indicating that, with the exception of some trajectories that cannot be salvaged, the LSTM improves the tracking capabilities of the method by reducing trajectory ambiguities, an assumption that is also supported by the fact that identity switches reduces significantly. Compared to the other Kalman-based methods, our proposed tracking method achieves higher results on all metrics, with the exception of MOTP, which is just <1.0% lower than OC-SORT.

The same performance gains can be observed for all models in the YOLOX family, with YOLOX-S achieving the best overall performance in MOTA score, while the larger models YOLOX-X and YOLOX-L obtain the best results in MOTP and IDF1 scores, respectively. For the MOTA index in particular, a careful analysis of Table 2 and Table 3 reveals that our model shows an improvement of 18.8% on average (±0.65% std, over all detectors) from the vanilla ByteTrack model, whereas it consistently outperforms SORT, DeepSort, MOTDT, OC-SORT, and StrongSORT, with average MOTA improvements of 10.43±1.66%, 28.16±1.74%, 51.82±8.49%, 14.47±2%, and 8.07±0.65%, respectively.

Another observation can be seen with careful tuning of the tracking threshold (see Section 4.1.2) on a per-video basis, which can potentially yield even higher results. This hyperparameter is a confidence measure, which determines that detections corresponding to high score bounding boxes will be considered for assignment to existing tracklets during the first association step. On the contrary, detections corresponding to lower scores will be taken into account by the second tracklet association step. By these means, a high tracking threshold might reject potential correct assignments during the first association. In this direction, we refer the reader to Appendix A.1 for an extensive ablative study on the effects of the various tracking threshold values on the final performance. Table A1 indicates that for relatively low tracking thresholds (i.e., 0.3 or 0.4), both competing systems (the vanilla tracker and our proposed ByteTrack+LSTM) with all possible backbone detector variants (i.e., YOLOX-X, …, YOLOX-Nano) regularly show improved performance and stable difference in the obtained accuracy.

Concerning the effect of the network architecture for the LSTM component, we note that from the ablation analysis presented in Appendix A.2, all possible network configurations (even a single layer with 128 hidden units) regularly perform well for all indices regardless of the underlying detector network. One possible explanation might be related to a relatively low number of consumer instances that need to tracked, in contrast with other multi-object tracking datasets, for which larger LSTM network architectures might seem more preferable to obtain increased performance at the cost of running speeds.

As far as practicality is concerned, in the *Consumers* dataset, the proposed method achieves competitive overall running speeds with other MOT methods, trailing the vanilla ByteTrack and SORT by <1–3 FPS, depending on the size of the detector used, while significantly outperforming both methods in all tracking metrics. Notably, the increased computational requirements of the LSTM lead to a negligible reduction in running speed, even in cases where multiple consumer trajectories need to be calculated. The overall FPS values obtained by our method for several backbone detection networks showcase its practical applications in real-time scenarios depending on the available resources.

As for the apparent age and gender estimation model, the one selected for use in the unified model is the Inception-based architecture with the attribute localization module, which achieves acceptable performance using just full body images in a challenging problem where face is an important factor in successful demographic attribute estimation. In our experiments, this model’s performance is competitive with other larger models, especially in the case where the raw data is used for training and the anonymized data for testing, a scenario which reflects real-world conditions. Anonymizing the images does not significantly affect performance, thereby confirming the method’s robustness in the absence or distortion of facial characteristics, an important feature for privacy-sensitive applications, regardless of the employed anonymization method. Finally, the small size and computational simplicity of this model enables its integration with the multi-object tracking method in the end-to-end visual tracking and apparent age/gender prediction pipeline for practical retail applications.

## 7. Conclusions

In this work, we introduced two novel datasets that can be used for a variety of tasks, such as (indoor) multi-object tracking or demographic attribute estimation. The datasets are collected from static cameras and depict indoor scenarios with good lighting conditions and many instances of occlusions. The *Consumers* dataset comprises video sequences of consumers, while the second one (*BID*) comprises cropped full-body images. To comply with data privacy regulations, any facial information was thoroughly removed. This means that potential missed anonymizations of the employed software (e.g., deface) were manually rectified. Furthermore, we provided extensive experiments on several recent works on both multi-object tracking and apparent age and gender estimation, thereby allowing fair comparisons among different approaches, potentially applicable to real-time scenarios that might favor a computer vision project’s needs. Moreover, the organization of both datasets follows a consistent sequence-by-sequence, frame-by-frame, and target-by-target structure. This design facilitates their use in various essential tasks, including but not limited to indoor person detection and re-identification.

Our proposed approach for consumer tracking wherein the LSTM module replaces traditional Kalman filters for the task of motion estimation showed significant performance improvements on our dataset, reflected by the reported numerical results. Finally, owing to their relatively simple architectures, both methods can be combined in an end-to-end system that still retains acceptable performance for deployment in real-world scenarios, as showcased in the example qualitative results.

Finally, our work aims to act as a both a theoretical and practical standpoint from which R&D can benefit in terms of selecting the appropriate methods and tools before developing a computer vision application for retail stores, able to scale in industrial level.

## Figures and Tables

**Table 1:** Different models of the YOLOX family produced by manipulating the width and depth factors that control the convolution layer channel dimensionality and bottlenecks in the model backbone and head. Parameters are measured in millions.

Model	Depth, Width	Parameters	GFLOPS
YOLOX-X	1.33, 1.25	99.1	281.9
YOLOX-L	1.00, 1.00	54.2	155.6
YOLOX-M	0.67, 0.75	25.3	73.8
YOLOX-S	0.33, 0.50	9.00	26.8
YOLOX-Tiny	0.33, 0.375	5.06	6.45
YOLOX-Nano ^1^	0.33, 0.25	0.91	1.08

^1^ The *Nano* model uses depthwise convolutions.

**Table 2:** Quantitative results of several MOT methods on the *Consumers* test set for YOLOX-X,L,M backbones. Our proposed association method is denoted in bold. ↑ means higher values are better. Measured FPS is the total runtime (detection and association) for all 25 sequences in the test set averaged across 5 runs on a single NVIDIA RTX2080 Ti GPU.

Backbone	Association	MOTA ↑	MOTP ↑	IDF1 ↑	MT ↑	PT	ML ↓	IDSw ↓	Avg. FPS ↑
YOLOX-X	ByteTrack	64.6%	82.8%	63.5%	24	22	3	91	18.41 ± 0.02
	**ByteTrack + LSTM**	**82.2%**	**90.3**%	**86.3%**	**35**	12	**2**	**8**	18.35 ± 0.01
	SORT	69.8%	83.5%	77.5%	22	23	4	12	**18.56 ± 0.02**
	DeepSORT	52.8%	76.4%	68.7%	14	31	4	13	9.22 ± 0.01
	MOTDT	41.8%	78.9%	58.8%	29	18	**2**	49	8.70 ± 0.01
	OC-SORT	65.0%	**90.3%**	74.5%	21	21	7	14	17.84 ± 0.43
	StrongSORT	74.4%	88.4%	82.2%	28	19	**2**	**8**	9.19 ± 0.01
YOLOX-L	ByteTrack	62.3%	82.3%	59.7%	25	21	**3**	109	29.52 ± 0.02
	**ByteTrack + LSTM**	**81.3%**	89.5%	**86.3%**	**35**	11	**3**	**5**	29.38 ± 0.02
	SORT	71.0%	82.6%	78.0%	22	22	5	11	**29.76 ± 0.04**
	DeepSORT	53.4%	76.1%	67.9%	17	28	4	14	11.46 ± 0.04
	MOTDT	26.0%	77.7%	53.3%	31	15	**3**	55	10.54 ± 0.05
	OC-SORT	66.1%	**89.8%**	76.0%	20	21	8	10	24.81 ± 0.01
	StrongSORT	72.9%	87.9%	80.9%	30	15	4	9	11.29 ± 0.06
YOLOX-M	ByteTrack	63.9%	81.2%	64.0%	22	23	4	94	42.88 ± 0.11
	**ByteTrack + LSTM**	**83.2%**	89.0%	**85.8%**	**34**	13	**2**	**5**	42.53 ± 0.11
	SORT	70.5%	82.1%	78.4%	24	23	**2**	11	**43.65 ± 0.15**
	DeepSORT	53.8%	75.6%	70.6%	16	31	**2**	11	12.89 ± 0.04
	MOTDT	25.8%	78.4%	54.0%	32	15	**2**	51	11.59 ± 0.03
	OC-SORT	66.8%	**89.2%**	75.9%	21	22	6	11	30.55 ± 0.05
	StrongSORT	74.2%	86.8%	83.8%	29	18	**2**	6	12.64 ± 0.03

**Table 3:** Quantitative results of several MOT methods on the *Consumers* test set for the more lightweight YOLOX backbones (“S”, “Tiny”, “Nano”). Our proposed association method is denoted in bold. ↑ means higher values are better. Measured FPS is the total runtime (detection and association) for all 25 sequences in the test set averaged across 5 runs on a single NVIDIA RTX2080 Ti GPU.

Backbone	Association	MOTA ↑	MOTP ↑	IDF1 ↑	MT ↑	PT	ML ↓	IDSw ↓	Avg. FPS ↑
YOLOX-S	ByteTrack	65.2%	81.3%	62.2%	26	21	**2**	115	72.82 ± 0.31
	**ByteTrack + LSTM**	**83.6%**	88.4%	**87.7%**	**37**	10	**2**	**6**	71.54 ± 0.25
	SORT	74.4%	82.8%	79.7%	26	21	**2**	17	**74.94 ± 0.28**
	DeepSORT	54.1%	76.4%	69.4%	16	31	**2**	22	14.73 ± <0.01
	MOTDT	26.5%	77.7%	53.2%	35	12	**2**	62	13.41 ± 0.02
	OC-SORT	70.9%	**88.7%**	78.9%	23	20	6	10	40.83 ± 0.22
	StrongSORT	75.0%	86.8%	80.9%	28	19	**2**	15	14.64 ± 0.01
YOLOX-Tiny	ByteTrack	63.1%	82.1%	65.5%	27	20	**2**	100	73.62 ± 0.18
	**ByteTrack + LSTM**	**82.0%**	88.4%	**88.0%**	**38**	9	**2**	**5**	72.62 ± 0.29
	SORT	74.0%	81.5%	81.3%	26	20	3	9	**75.63 ± 0.49**
	DeepSORT	53.7%	77.1%	70.8%	21	25	3	11	14.70 ± <0.01
	MOTDT	21.0%	77.5%	52.3%	31	15	3	55	13.30 ± 0.02
	OC-SORT	70.5%	**88.9%**	79.0%	24	19	6	9	41.03 ± 0.51
	StrongSORT	74.7%	86.5%	84.0%	34	13	**2**	**5**	14.60 ± 0.01
YOLOX-Nano	ByteTrack	62.3%	80.9%	63.0%	26	20	3	105	67.01 ± 0.13
	**ByteTrack + LSTM**	**81.9%**	86.8%	**87.3%**	**38**	9	**2**	**6**	66.21 ± 0.20
	SORT	71.9%	81.4%	80.8%	26	19	4	9	**68.39 ± 0.19**
	DeepSORT	57.4%	75.8%	71.8%	20	24	5	15	14.47 ± <0.01
	MOTDT	42.2%	77.2%	58.3%	30	15	4	52	13.18 ± 0.02
	OC-SORT	68.1%	**86.9%**	77.6%	23	21	5	7	35.16 ± 0.12
	StrongSORT	74.6%	85.3%	83.2%	32	13	4	7	14.31 ± 0.03

**Table 4:** Numerical results of several age and gender estimation methods trained and evaluated on the raw *BID* dataset. This setup evaluates the theoretical effectiveness of the methods with raw data. The method used for estimation in the end-to-end system is highlighted. ↑ indicates that higher is better.

Method	Backbone	mA ↑	Accuracy ↑	Precision ↑	Recall ↑	F1 Score ↑
**ALM** [111]	**Inception**	0.7770	0.6630	0.6845	0.7905	0.7337
MSSC [123]	ResNet50	0.7266	0.6218	0.6436	0.7482	0.6783
SOLIDER [124]	Swin-Base	0.7733	0.6265	0.6668	0.6658	0.6663
	Swin-Small	0.7444	0.6010	0.6389	0.6420	0.6404
	Swin-Tiny	0.7354	0.6104	0.6545	0.6539	0.6542
ROP [125]	ResNet50	0.7003	0.6201	0.6652	0.6565	0.6608
	ViT-Base	0.7323	0.6468	0.6767	0.6936	0.6850
	ViT-Small	0.7073	0.6301	0.6571	0.6790	0.6679

**Table 5:** Numerical results of several age and gender estimation methods trained **and** evaluated on the anonymized *BID* dataset using the *deface* software. This experiment evaluates the robustness of the method when trained and tested on anonymous data to ensure that blurring the face area does not significantly affect performance. The method used for estimation in the end-to-end system is highlighted. ↑ indicates that higher is better.

Method	Backbone	mA ↑	Accuracy ↑	Precision ↑	Recall ↑	F1 Score ↑
**ALM** [111]	**Inception**	0.6997	0.6248	0.6597	0.7001	0.6793
MSSC [123]	ResNet50	0.6788	0.5600	0.5924	0.7621	0.6459
SOLIDER [124]	Swin-Base	0.7155	0.6258	0.6448	0.6839	0.6637
	Swin-Small	0.7395	0.6282	0.6674	0.6636	0.6655
	Swin-Tiny	0.7558	0.5874	0.6323	0.6477	0.6399
ROP [125]	ResNet50	0.7015	0.6227	0.6534	0.6680	0.6606
	ViT-Base	0.6934	0.5966	0.6240	0.6530	0.6382
	ViT-Small	0.6957	0.6036	0.6395	0.6561	0.6477

**Table 6:** Numerical results of several age and gender estimation methods trained on the raw *BID* dataset and evaluated on the *BID* test set after anonymization using the *deface* software. This final experiment resembles real-world conditions where the method is trained using raw data to obtain a good set of weights and then deployed in applications, and therefore, inference might be performed on anonymized data. The method used for estimation in the end-to-end system is highlighted. ↑ indicates that higher is better.

Method	Backbone	mA ↑	Accuracy ↑	Precision ↑	Recall ↑	F1 Score ↑
**ALM** [111]	**Inception**	0.7639	0.6457	0.6771	0.7588	0.7156
MSSC [123]	ResNet50	0.7060	0.5836	0.6091	0.7031	0.6389
SOLIDER [124]	Swin-Base	0.7539	0.6096	0.6539	0.6495	0.6517
	Swin-Small	0.7295	0.5797	0.6201	0.6213	0.6207
	Swin-Tiny	0.7265	0.5837	0.6299	0.6279	0.6289
ROP [125]	ResNet50	0.6968	0.5869	0.6321	0.6376	0.6348
	ViT-Base	0.6887	0.6063	0.6384	0.6601	0.6491
	ViT-Small	0.6730	0.5722	0.6182	0.6168	0.6175

**Table 7:** Ablative analysis of the reduction ratio in the attention network used by the attribute localization module of the apparent age/gender estimation method (Inception backbone). Training is performed on the raw *BID* dataset, while testing uses the anonymized test set. ↑ indicates that higher is better.

Attention Method	mA ↑	Accuracy ↑	Precision ↑	Recall ↑	F1 Score ↑
SE (reduction ratio = 8)	0.6996	0.5692	0.6090	0.6609	0.6339
SE (reduction ratio = 16)	0.7639	0.6457	0.6771	0.7588	0.7156
SE (reduction ratio = 32)	0.7051	0.5806	0.6194	0.6873	0.6516
SB (reduction ratio = 8)	0.7316	0.5965	0.6277	0.7169	0.6690
SB (reduction ratio = 16)	0.7225	0.6019	0.6284	0.7191	0.6707
SB (reduction ratio = 32)	0.7037	0.6102	0.6328	0.7125	0.6703

## Data Availability

The data presented in this study are available on https://www.kaggle.com/datasets/angelosgiotis/consumers-bid. Code and pre-trained weights related to our implementation are available on https://github.com/jpanagos/consumers-bid.

## Appendix A. Additional Tracking Method Ablations

### Appendix A.1. Tracking Threshold Analysis

**Table A1:** Analysis of the effect of the tracking threshold in the final result. We compare our method (in bold) with the vanilla ByteTrack [44] on our *Customers* dataset. ↑ indicates that higher is better.

Backbone (Association Method)	Tracking Threshold	MOTA ↑	MOTP ↑	IDF1 ↑
YOLOX-X (ByteTrack)	0.6	63.8%	82.7%	63.7%
	0.5	64.0%	82.8%	64.1%
	0.4	63.8%	82.9%	63.6%
	0.3	64.3%	82.7%	63.6%
YOLOX-X (**ByteTrack + LSTM**)	0.6	80.9%	90.4%	84.5%
	0.5	81.0%	90.4%	84.4%
	0.4	82.2%	90.3%	86.3%
	0.3	82.2%	90.3%	86.3%
YOLOX-L (ByteTrack)	0.6	59.1%	83.0%	57.6%
	0.5	60.1%	82.5%	58.1%
	0.4	60.7%	82.6%	58.4%
	0.3	62.3%	82.6%	59.4%
YOLOX-L (**ByteTrack + LSTM**)	0.6	75.8%	89.7%	82.7%
	0.5	77.4%	89.6%	84.7%
	0.4	78.4%	89.5%	85.8%
	0.3	81.6%	89.5%	88.1%
YOLOX-M (ByteTrack)	0.6	63.2%	82.3%	63.5%
	0.5	63.9%	82.2%	62.9%
	0.4	64.1%	81.9%	62.7%
	0.3	63.8%	81.7%	62.2%
YOLOX-M (**ByteTrack + LSTM**)	0.6	79.5%	89.2%	84.4%
	0.5	80.9%	89.2%	85.1%
	0.4	82.3%	89.1%	86.2%
	0.3	83.2%	89.0%	85.8%
YOLOX-S (ByteTrack)	0.6	63.3%	81.4%	62.6%
	0.5	64.7%	81.4%	62.5%
	0.4	65.0%	81.3%	62.4%
	0.3	65.2%	81.3%	62.2%
YOLOX-S (**ByteTrack + LSTM**)	0.6	81.5%	88.5%	86.7%
	0.5	83.0%	88.5%	87.4%
	0.4	83.6%	88.4%	87.7%
	0.3	83.4%	88.4%	87.3%
YOLOX-Tiny (ByteTrack)	0.6	62.3%	82.3%	64.2%
	0.5	63.0%	82.2%	65.1%
	0.4	63.1%	82.0%	65.3%
	0.3	63.2%	82.1%	65.5%
YOLOX-Tiny (**ByteTrack + LSTM**)	0.6	78.1%	88.8%	85.9%
	0.5	80.4%	88.6%	87.2%
	0.4	80.2%	88.5%	86.8%
	0.3	81.8%	88.4%	87.7%
YOLOX-Nano (ByteTrack)	0.6	60.3%	80.9%	61.5%
	0.5	61.6%	80.9%	61.6%
	0.4	61.4%	81.1%	61.9%
	0.3	61.8%	80.9%	61.8%
YOLOX-Nano (**ByteTrack + LSTM**)	0.6	77.3%	87.1%	82.1%
	0.5	79.9%	86.9%	85.5%
	0.4	80.3%	86.9%	85.4%
	0.3	81.9%	86.8%	87.3%

### Appendix A.2. LSTM Configuration Analysis

**Table A2:** LSTM configuration analysis on the *Consumers* test sequences. In all these experiments, the association method used is ByteTrack and motion estimation is handled by the LSTM. ↑ indicates that higher is better.

Backbone	LSTM Config (Layers × Neurons)	MOTA ↑	MOTP ↑	IDF1 ↑	Avg. FPS ↑
YOLOX-X	1×64	82.2%	90.4%	85.5%	18.25 ± 0.02
	1×128	82.2%	90.3%	86.3%	18.27 ± 0.01
	1×256	82.1%	90.3%	86.2%	18.30 ±<0.01
	1×512	82.1%	90.3%	86.2%	18.28 ±<0.01
	1×1024	82.2%	90.3%	86.3%	18.29 ±<0.01
	2×64	82.2%	90.3%	86.3%	18.27 ±<0.01
	2×128	82.1%	90.3%	86.2%	18.28 ±<0.01
	2×256	82.1%	90.3%	86.2%	18.28 ±<0.01
	2×512	82.2%	90.4%	86.1%	18.25 ± 0.01
YOLOX-L	1×64	81.3%	89.5%	86.3%	28.96 ± 0.03
	1×128	81.6%	89.5%	88.1%	28.97 ± 0.05
	1×256	81.5%	89.5%	88.0%	28.99 ± 0.06
	1×512	81.5%	89.5%	88.0%	28.98 ± 0.06
	1×1024	81.7%	89.5%	88.1%	28.99 ± 0.05
	2×64	81.5%	89.5%	88.0%	28.96 ± 0.02
	2×128	81.5%	89.5%	88.0%	28.96 ± 0.02
	2×256	81.4%	89.5%	88.0%	28.94 ± 0.02
	2×512	81.5%	89.5%	88.0%	28.88 ± 0.04
YOLOX-M	1×64	82.9%	89.0%	85.0%	41.60 ± 0.03
	1×128	83.2%	89.0%	85.8%	41.75 ± 0.05
	1×256	83.1%	89.0%	85.9%	41.68 ± 0.09
	1×512	83.2%	89.0%	85.8%	41.63 ± 0.08
	1×1024	83.2%	89.0%	85.8%	41.59 ± 0.11
	2×64	83.1%	89.0%	85.9%	41.60 ± 0.07
	2×128	83.1%	89.0%	85.9%	41.64 ± 0.06
	2×256	82.9%	89.0%	85.8%	41.51 ± 0.05
	2×512	83.1%	89.0%	86.6%	41.49 ± 0.09
YOLOX-S	1×64	83.5%	88.4%	86.7%	59.50 ± 0.10
	1×128	83.6%	88.4%	87.7%	59.79 ± 0.19
	1×256	83.6%	88.4%	87.7%	59.64 ± 0.14
	1×512	83.6%	88.4%	87.7%	59.54 ± 0.18
	1×1024	83.4%	88.4%	87.8%	59.43 ± 0.20
	2×64	83.5%	88.4%	87.6%	59.27 ± 0.22
	2×128	83.6%	88.4%	87.7%	59.23 ± 0.19
	2×256	83.5%	88.4%	87.6%	59.36 ± 0.16
	2×512	83.5%	88.4%	87.6%	59.35 ± 0.25
YOLOX-Tiny	1×64	81.8%	88.5%	87.3%	60.34 ± 0.11
	1×128	81.8%	88.4%	87.7%	60.52 ± 0.35
	1×256	82.0%	88.4%	87.8%	60.35 ± 0.17
	1×512	81.8%	88.4%	87.7%	60.11 ± 0.12
	1×1024	81.9%	88.4%	87.8%	60.30 ± 0.24
	2×64	82.0%	88.4%	88.0%	60.03 ± 0.23
	2×128	82.0%	88.4%	88.0%	60.11 ± 0.09
	2×256	81.8%	88.5%	87.9%	60.16 ± 0.16
	2×512	81.8%	88.4%	87.7%	60.21 ± 0.11
YOLOX-Nano	1×64	81.6%	86.8%	86.1%	54.60 ± 0.19
	1×128	81.9%	86.8%	87.3%	54.78 ± 0.30
	1×256	81.7%	86.8%	87.2%	54.70 ± 0.11
	1×512	81.8%	86.4%	87.3%	54.66 ± 0.09
	1×1024	81.8%	86.4%	87.3%	54.81 ± 0.22
	2×64	81.8%	86.4%	87.3%	54.57 ± 0.09
	2×128	81.8%	86.4%	87.3%	54.37 ± 0.17
	2×256	81.8%	86.4%	87.3%	54.53 ± 0.11
	2×512	81.8%	86.4%	87.3%	54.38 ± 0.22

## Appendix B. Additional Age/Gender Estimation Method Experiments

**Table A3:** Numerical results of several age and gender estimation methods trained and evaluated on the anonymized *BID* dataset using the *deep-privacy* method which replaces faces instead of applying a blurring filter. The method used for estimation in the end-to-end system is highlighted. ↑ indicates that higher is better.

Method	Backbone	mA ↑	Accuracy ↑	Precision ↑	Recall ↑	F1 Score ↑
**ALM** [111]	**Inception**	0.7256	0.6457	0.6678	0.7566	0.7094
MSSC [123]	ResNet50	0.6339	0.5342	0.5656	0.7027	0.6098
SOLIDER [124]	Swin-Base	0.7568	0.6245	0.6809	0.6623	0.6715
	Swin-Small	0.7600	0.6296	0.6595	0.6772	0.6682
	Swin-Tiny	0.7255	0.6165	0.6596	0.6649	0.6622
ROP [125]	ResNet50	0.7063	0.6327	0.6728	0.6795	0.6761
	ViT-Base	0.7452	0.6482	0.6849	0.7019	0.6933
	ViT-Small	0.6997	0.6171	0.6565	0.6772	0.6667

**Table A4:** Numerical results of several age and gender estimation methods trained on the raw *BID* dataset and evaluated on the deep-privacy-anonymized test set. The method used for estimation in the end-to-end system is highlighted. ↑ indicates that higher is better.

Method	Backbone	mA ↑	Accuracy ↑	Precision ↑	Recall ↑	F1 Score ↑
**ALM** [111]	**Inception**	0.7363	0.6272	0.6577	0.7420	0.6973
MSSC [123]	ResNet50	0.7023	0.5779	0.6064	0.6946	0.6337
SOLIDER [124]	Swin-Base	0.7497	0.6092	0.6496	0.6556	0.6526
	Swin-Small	0.7309	0.5935	0.6337	0.6340	0.6339
	Swin-Tiny	0.7230	0.5968	0.6424	0.6433	0.6429
ROP [125]	ResNet50	0.6957	0.5944	0.6398	0.6486	0.6441
	ViT-Base	0.6964	0.6091	0.6415	0.6601	0.6507
	ViT-Small	0.6964	0.6075	0.6509	0.6517	0.6513
